# Exacerbation of Thrombotic Responses to Silver Nanoparticles in Hypertensive Mouse Model

**DOI:** 10.1155/2022/2079630

**Published:** 2022-01-15

**Authors:** Zannatul Ferdous, Sumaya Beegam, Nur E. Zaaba, Ozaz Elzaki, Saeed Tariq, Yaser E. Greish, Badreldin H. Ali, Abderrahim Nemmar

**Affiliations:** ^1^Department of Physiology, College of Medicine and Health Sciences, United Arab Emirates University, P.O. Box 17666, Al Ain, UAE; ^2^Department of Anatomy, College of Medicine and Health Science, United Arab Emirates University, P.O. Box 17666, Al Ain, UAE; ^3^Department of Chemistry, College of Sciences, United Arab Emirates University, P.O. Box 17666, Al Ain, UAE; ^4^Department of Pharmacology and Clinical Pharmacy, Sultan Qaboos University, P.O. Box 35, Muscat 123, Al-Khod, Oman; ^5^Zayed Center for Health Sciences, United Arab Emirates University, UAE

## Abstract

With advent of nanotechnology, silver nanoparticles, AgNPs owing majorly to their antibacterial properties, are used widely in food industry and biomedical applications implying human exposure by various routes including inhalation. Several reports have suggested AgNPs induced pathophysiological effects in a cardiovascular system. However, cardiovascular diseases such as hypertension may interfere with AgNPs-induced response, yet majority of them are understudied. The aim of this work was to evaluate the thrombotic complications in response to polyethylene glycol- (PEG-) coated AgNPs using an experimental hypertensive (HT) mouse model. Saline (control) or PEG-AgNPs (0.5 mg/kg) were intratracheally (i.t.) instilled four times, i.e., on days 7, 14, 21, and 28 post-angiotensin II-induced HT, or vehicle (saline) infusion. On day 29, various parameters were assessed including thrombosis in pial arterioles and venules, platelet aggregation in whole blood in vitro, plasma markers of coagulation, and fibrinolysis and systemic oxidative stress. Pulmonary exposure to PEG-AgNPs in HT mice induced an aggravation of *in vivo* thrombosis in pial arterioles and venules compared to normotensive (NT) mice exposed to PEG-AgNPs or HT mice given saline. The prothrombin time, activated partial thromboplastin time, and platelet aggregation in vitro were exacerbated after exposure to PEG-AgNPs in HT mice compared with either NT mice exposed to nanoparticles or HT mice exposed to saline. Elevated concentrations of fibrinogen, plasminogen activator inhibitor-1, and von Willebrand factor were seen after the exposure to PEG-AgNPs in HT mice compared with either PEG-AgNPs exposed NT mice or HT mice given with saline. Likewise, the plasma levels of superoxide dismutase and nitric oxide were augmented by PEG-AgNPs in HT mice compared with either NT mice exposed to nanoparticles or HT mice exposed to saline. Collectively, these results demonstrate that PEG-AgNPs can potentially exacerbate the in vivo and in vitro procoagulatory and oxidative stress effect in HT mice and suggest that population with hypertension are at higher risk of the toxicity of PEG-AgNPs.

## 1. Introduction

Silver nanoparticles (AgNPs) became one of the most investigated engineered nanomaterials during the past few years, given the fact that these nanomaterials proved to have interesting, challenging, and promising characteristics suitable for various household and biomedical applications [1–3]. The widespread application in turn results in environmental contamination and human exposure raising serious concern about their potential adverse effects and toxicity on human health [2, 4]. Of all the various routes of exposure of nanoparticles reported so far, pulmonary exposure provides a major a potential route to aerosolized AgNPs used in health sprays, nebulizers, deodorants, and disinfectants [5]. Moreover, inhalation exposure to these particles is inescapable to workers in nanosilver-manufacturing industries, particularly during particle synthesis and handling of dry powders, as well as during the manufacture of AgNPs-containing products [6].

A collection of studies have previously addressed the effects and applications of different kinds of AgNPs (shaped, sized, coated and functionalized) in several components of the cardiovascular system, such as endothelial cells, isolated vessels, and organs as well as integrative animal models, trying to elucidate the underlying mechanisms involved in their pathophysiological effect and hence to understand their implication in the field of biomedicine [7–11]. For instance, Sun et al. [12] demonstrated that AgNPs exposure significantly and dose-dependently decreased the cell viability, induced NADPH oxidase 4 and nuclear factor erythroid 2-related factor 2 (Nrf2) mediated oxidative stress, and led to early apoptosis in human umbilical vein endothelial cells. Using Langendorff rat heart preparation, Ramirez-Lee and colleagues evaluated direct actions of AgNPs (15 ± 4 nm) on coronary vascular tone and cardiac contractility [13]. Similarly, we have recently demonstrated significant pathophysiological effect to pulmonary-exposed polyvinylpyrrolidone and citrate-coated AgNPs (10 nm) on a cardiovascular system particularly on the thrombotic events, oxidative stress, inflammatory markers, DNA damage, and apoptosis [14]. Our *in vitro* study further revealed the potential of these same nanoparticles to cause significant erythrocytic oxidative damage and eryptosis [10].

Although the number of evidence about the toxic effects and mechanisms induced by AgNPs on heart is limited, there are much fewer investigations on the impeding effects of these nanoparticles on populations with cardiovascular pathologies such as hypertension. In this regard, previous studies have demonstrated that pulmonary exposure to engineered nanomaterials is capable of aggravating cardiovascular dysfunction via mechanisms including systemic inflammation, coronary artery dysfunction, metabolic derangement, autonomic dysregulation, and oxidative stress [15–18]. Oxidative stress contributed by increased reactive oxygen species level generates an imbalance between reactive oxygen species generation and antioxidant defence mechanism such as catalase and superoxide dismutase [19]. This process also contributes to the development of a common cardiovascular disorder, namely, hypertension [20]. In addition, evidence for the prothrombotic and hypercoagulable state in hypertension has been extensively reviewed [20–22]. In fact, some studies have reported abnormalities in the coagulation and fibrinolytic pathways, as well as in platelets and the endothelium, among hypertensive experimental models and patients [23–25]. In this context, we have recently shown significant oxidative stress and prothrombotic and inflammatory effects in mice exposed to 10 nm AgNPs via intratracheal instillation [14]. Nevertheless, influence of hypertension on the latter effects has not been studied so far.

AgNPs are coated with various natural or synthetic polymers in order to preserve their bioavailability, increase stability, and reduce toxicity [2, 3, 26]. In this regard, polyethylene glycol (PEG) has been widely applied as an effective stabilizing agent in the fabrication of AgNPs and other metal nanoparticles. Consequently, the aim of this study is to assess the effect of PEG-AgNPs, in a mouse model of angiotensin (ANG) II-induced hypertension, on thrombotic events, coagulation profile, and oxidative stress by measuring thrombotic occlusion time in pial arterioles and venules, prothrombin time (PT), activated partial thromboplastin time (aPTT), fibrinogen, plasminogen activator inhibitor-1 (PAI-1), von Willebrand factor (vWF), superoxide dismutase, and total nitric oxide.

## 2. Materials and Methods

### 2.1. Nanoparticles

Suspensions of polyethylene glycol- (PEG-) coated silver nanoparticles (PEG-AgNPs) of 40.6 ± 3.8 nm (BioPure™) were purchased from NanoComposix (San Diego, CA, USA). The provided stock concentrations were 1.0 mg/ml with silver purity of 99.99% and endotoxin level < 2.5 EU/ml. The nanoparticle surface area was 13.8 m2/g. PEG-AgNPs were suspended in sterile 0.9% NaCl. Silver acetate (AgAc), as the source of Ag^+^ ions, purchased from Sigma-Aldrich (#216674, St. Louis, MO, USA), was dissolved in sterile water to yield a stock concentration of 1 mg/ml. In order to reduce nanoparticle aggregation, the suspensions of PEG-AgNPs were constantly sonicated (Clifton Ultrasonic Bath, Clifton, NJ, USA) for 10 min and vortexed prior their dilution and intratracheal (i.t.) instillation.

### 2.2. Animal, Experimental Hypertensive Model, and Dosing

Both male and female BALB/C mice of age 8–10 weeks, weighing 20 to 25 g (Animal House of the College of Medicine and Health Sciences, United Arab Emirates University), were housed in light- (12 h light : 12 h dark cycle) and temperature-controlled (22 ± 1°C) rooms. They had free access to commercial laboratory chow and were provided tap water *ad libitum*.

A well-validated murine model of hypertension (HT mice) was utilized [21, 27, 28]. BALB/c (8-10 weeks old) mice were administered angiotensin II (ANG II, 0.75 mg/kg/day in 0.15 mol/l NaCl and 0.01 N acetic acid) or vehicle (normotensive (NT), i.e., control mice) for the entire duration of the experiments using an osmotic pump (Alzet osmotic pump model 2006, Durect Corporation, Cupertino, CA, USA). This treatment delivers ANG II plasma concentration equivalent to that observed in patients with renovascular hypertension [28, 29]. The systolic blood pressure (SBP) was measured using computerized noninvasive tail cuff manometry system (ADInstruments, Colorado Springs, USA). SBP was recorded prior to the measurement of thrombosis or animal sacrifice for blood collection and analysis.

Pulmonary exposure was achieved by intratracheal (i.t.) instillation [30, 31]. Mice were anesthetized with isoflurane and positioned supine with an extended neck on an angled board. A Becton Dickinson 24 Gauge cannula was introduced via the mouth into the trachea. The PEG-AgNPs (0.5 mg/kg) or saline (control) was instilled (100 *μ*l) via a sterile syringe, followed by an equal volume of air bolus. The nanoparticles or saline were i.t. instilled four times, i.e., on days 7, 14, 21, and 28 post-ANG II or vehicle (control) infusion. Another similar group of NT and HT mice received AgAc as a source of Ag^+^. On day 29, mice weights were taken, and various cardiovascular parameters were assessed.

This study was reviewed and approved (approval # ERA_2019_5876) by the United Arab Emirates University Animal Ethics Committee, and experiments were performed in accordance with protocols approved by the committee.

### 2.3. Characterization of PEG-AgNPs

Transmission electron microscopy (TEM) of AgNPs was performed by a method described in our previous paper [10]. Briefly, the suspensions were subjected to sonication at room temperature for 15 min prior to processing for TEM. A drop of PEG-AgNPs suspensions was deposited on a 200-mesh Formvar/Carbon coated copper grid and allowed to dry for 1 h at room temperature. Then, the grids were examined and photographed at different magnifications using Tecnai™ G^2^ Spirit transmission microscope (FEI Company, Hillsboro, OR, USA).

Regarding zeta potential analysis, the PEG-AgNPs suspensions were diluted to 10% by volume in absolute ethanol, vortexed for 10 minutes, and then were subjected to the size distribution and zeta potential measurements using Malvern zetasizer instrument (Malvern Panalytical, UK) and Zetasizer 7.11 software for the measurement and data processing. All measurements were carried out at room temperature and were done in triplicate.

### 2.4. Experimental Pial Arteriole and Venule Thrombosis Model

In separate animals, *in vivo* thrombogenesis in the pial arterioles and venules was assessed in NT and HT mice after saline or PEG-AgNPs or Ag^+^ ion exposure, according to a previously described technique [32]. Briefly, the animal was anesthetized with urethane (1 mg/g BW, i.p.), the trachea was intubated, and the right jugular vein was cannulated with a 2F venous catheter (Portex, Hythe, UK) for the administration of fluorescein (Sigma-Aldrich, St. Louis, MO, USA). Thereafter, craniotomy was first performed on the right temporoparietal cortex with a hand-held microdrill, and the dura was stripped open. Only untraumatized preparations were used, and those showing trauma to either microvessels or underlying brain tissue were discarded. Cerebral microcirculation was directly visualized using a fluorescence microscope (Olympus, Melville, NY, USA) connected to a camera and DVD recorder. A heating pad was used, and body temperature was raised to 37°C, as monitored by a rectal thermoprobe connected to a temperature reader (Physitemp Instruments, NJ, USA). A field containing arterioles and venules (15-20 *μ*m) in diameter was chosen. Such a field was taped prior to and during the photochemical insult, which was carried out by injecting fluorescein (0.1 ml/mouse of 5% solution) via the jugular vein, which was allowed to circulate for 30-40 sec. The cranial preparation was then exposed to stabilized mercury light. The photochemically induced injury to arterioles and venules, in turn, causes platelets to adhere at the site of endothelial damage and aggregate. Platelet aggregates and thrombus formation grow in size until complete vascular occlusion. The time from the injury until complete vascular occlusion (time to flow stop) in arterioles and venules was measured in seconds. At the end of the experiments, the animals were euthanized by an overdose of urethane.

### 2.5. Prothrombin Time (PT) and Activated Partial Thromboplastin Time (aPTT) Measurements in Plasma

The PT and aPTT were measured in plasma collected from treated mice by using TEClot PT-S and TEClot aPTT-S kits (TECO GmbH, Dieselstr. 1, 84088, Neufahrn, NB, Germany), according to the manufacturer's instruction. Briefly, the PT and aPTT were measured in platelet poor plasma (PPP), preincubated at 37°C for 3 minutes, followed by addition of PT and aPTT reagent, using a Merlin coagulometer (MC 1 VET, Merlin, Lemgo, Germany).

### 2.6. In Vitro Platelet Aggregation in Mouse Whole Blood

In vitro platelet aggregation in whole blood collected from NT or HT mice after i.t. instillation of saline or PEG-AgNPs or Ag^+^ ions was performed with slight modifications as previously described [32]. After anesthesia, blood from untreated mice was withdrawn from the inferior vena cava, placed in citrate (3.2%), and 0.1 ml aliquots were added to the well of a Merlin coagulometer (MC 1 VET; Merlin, Lemgo, Germany). Blood samples were incubated at 37.2°C with ADP (0.1 *μ*M) for 3 min and then stirred for another 3 min. At the end of this period, 25 *μ*l samples were removed and fixed on ice in 225 ml cellFix (Becton Dickinson, Franklin Lakes, NJ). After fixation, single platelets were counted in a VET ABX Micros with a mouse card (ABX). The occurrence of platelet aggregation induced by ADP caused a decrease in the counted single platelets in the blood (fall in the number of single platelets counted) obtained from the four studied groups compared with each other and with untreated (without ADP) whole blood obtained from control (unexposed) mice.

### 2.7. Measurement of Systemic Markers of Coagulation, Fibrinolysis, and vWF

The concentrations of fibrinogen (Molecular Innovation, Southfield, MI, USA) and plasminogen activation inhibitor (PAI-1, Molecular Innovation, Southfield, USA) were determined using an ELISA Kit. The plasma concentration of vWF (Molecular Innovation, Southfield, MI, USA) was measured using an ELISA kit.

### 2.8. Oxidative Stress Evaluation: Total NO_2_ and Superoxide Dismutase (SOD)

The determination of NO was performed with a total NO assay kit from R&D Systems (Minneapolis, MN, USA) which measures the more stable NO metabolites NO_2_^−^ and NO_3_^−^ [33]. SOD activity was measured as the conversion of nitroblue tetrazolium (NBT) to NBT-diformazan according to the vendor's protocol (Chemical Cayman, MI, USA). The extent of reduction in the appearance of NBT-formazan was used as a measure of SOD activity present in the plasma.

### 2.9. Statistics

All data are presented as the mean ± standard error of the mean, and the statistical significance was determined by one-way analysis of variance (ANOVA-1) followed by the Holm-Sidak post hoc test. *P* values less than 0.05 were regarded as significant using GraphPad Prism Ver. 5.01 (GraphPad Software Inc., La Jolla, CA, USA).

## 3. Results

### 3.1. Characterization of PEG-AgNPs and Establishment of Hypertension

**Figure 1:**
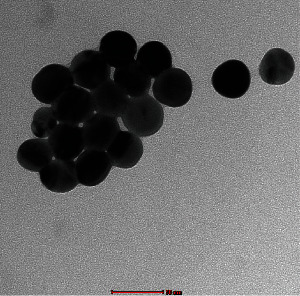
Transmission electron microscope (TEM) image of polyethylene glycol silver nanoparticles (PEG-AgNPs).

The morphology and particle size of PEG-AgNPs were determined by TEM are shown in Figure 1. TEM analysis of PEG-AgNPs revealed a homogenous particle size of approximately 40 nm in diameter, and this corroborates the size provided by the manufacturer. The nanoparticles were spherical in shape. The zeta potential assessments of PEG-AgNPs revealed that they were electroneutral (0.160 mV).

Mice infused with Angiotensin II exhibited a significant increase in SBP (*P* < 0.0001) compared with normotensive mice as shown in supplementary Figure S1.

### 3.2. Effect of PEG-AgNPs on Photochemically Induced Thrombosis in Pial Arterioles and Venules of Mice *In Vivo*

**Figure 2:**
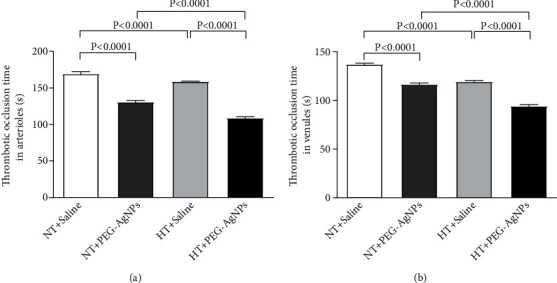
Thrombotic occlusion time in pial arterioles (a) or venules (b) following intratracheal instillation of saline or polyethylene glycol silver nanoparticles (PEG-AgNPs) in normotensive (NT) or hypertensive (HT) mice. Data are the mean ± SEM (*n* = 6–8 in each group). Statistical analysis by one-way ANOVA followed by Holm-Sidak's multiple comparison test.

The effect of PEG-AgNPs on thrombotic occlusion time is illustrated in Figure 2. PEG-AgNPs induced significant shortening of the thrombotic occlusion time (*P* < 0.0001) in both the arterioles (Figure 2(a)) and venules (Figure 2(b)) of HT mice compared to NT mice. Moreover, thrombotic occlusion time was significantly reduced in HT mice exposed to PEG-AgNPs (*P* < 0.0001) compared to HT mice exposed to saline.

Ag ions also caused significant shortening of thrombotic occlusion time (*P* < 0.0001) in NT and HT mice compared to controls receiving saline (data shown in supplementary Figure S2).

### 3.3. Effect of PEG-AgNPs on PT and aPTT

**Figure 3:**
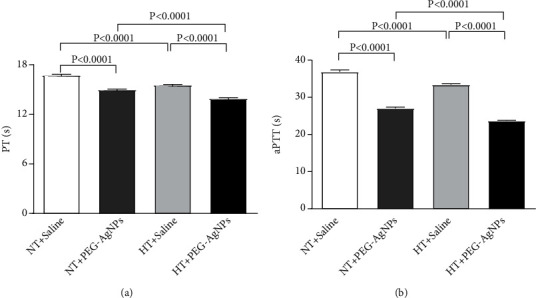
Prothrombin time (PT (a)) and activated partial thromboplastin time (aPTT (b)) measured following intratracheal instillation of saline or polyethylene glycol silver nanoparticles (PEG-AgNPs) in normotensive (NT) or hypertensive (HT) mice. Data are the mean ± SEM (*n* = 6–8 in each group). Statistical analysis by one-way ANOVA followed by Holm-Sidak's multiple comparison test.

Figure 3 represents the PT and aPTT in PPP collected from NT and HT mice treated with either saline or PEG-AgNPs. Compared to NT mice treated with PEG-AgNPs, a significant decrease in PT was observed in HT mice (*P* < 0.0001) exposed to PEG-AgNPs. Similar significant decrease was also observed with aPTT in HT mice exposed to PEG-AgNPs compared to NT mice given PEG-AgNPs. In addition, significant decreases in PT and aPTT were observed in HT mice exposed to PEG-AgNPs (*P* < 0.0001) compared to saline exposed HT mice. Likewise, there was a significant shortening of PT and aPTT in NT mice exposed to PEG-AgNPs (*P* < 0.0001) compared to NT mice treated with saline (Figure 3).

Similar significant reduction (*P* < 0.0001) in PT and aPTT was also observed in Ag^+^ ion-exposed NT and HT mice compared to their respective controls. Significant reduction (*P* < 0.0001) was also observed in Ag^+^ ion-exposed HT mice compared to NT mice (data shown in supplementary Figure S3).

### 3.4. Effect of PEG-AgNPs on Platelet Aggregation in Whole Blood In Vitro

**Figure 4:**
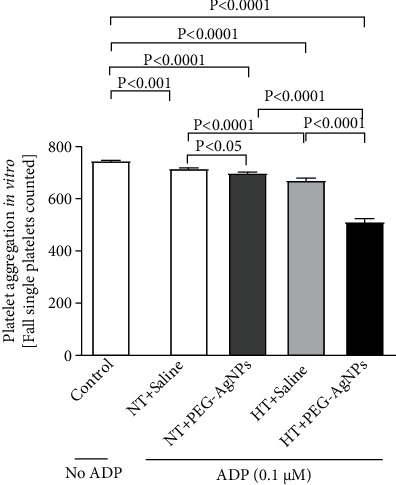
In vitro platelet aggregation in whole blood collected from normotensive (NT) or hypertensive (HT) mice after intratracheal (i.t.) instillation of saline or polyethylene glycol silver nanoparticles (PEG-AgNPs). Blood samples obtained from the aforementioned groups were incubated at 37°C with ADP (0.1 *μ*M) for 3 min and stirred for another 3 min, and single platelets were then counted. The degree of platelet aggregation in HT or NT mice exposed to PEG-AgNPs or saline was compared with each other and with that obtained in untreated (without ADP) whole blood obtained from control (unexposed) mice. Data are the mean ± SEM (*n* = 4). Statistical analysis by one-way ANOVA followed by Holm-Sidak's multiple comparison test.

Figure 4 illustrates the effect of e PEG-AgNPs on platelet aggregation in whole blood. The *in vitro* ADP incubation of whole blood collected from HT mice exposed to PEG-AgNPs caused a significant platelet aggregation compared with HT mice exposed to saline (*P* < 0.0001) and NT mice exposed to nanoparticles (*P* < 0.0001).

Ag^+^ ions also caused significant platelet aggregation in HT mice compared to the saline-treated HT group (*P* < 0.0001) and also NT mice exposed to Ag^+^ ions (*P* < 0.0001) (data shown in supplementary Figure S4).

### 3.5. Effect of PEG-AgNPs on Fibrinogen, PAI-1, and vWF

**Figure 5:**
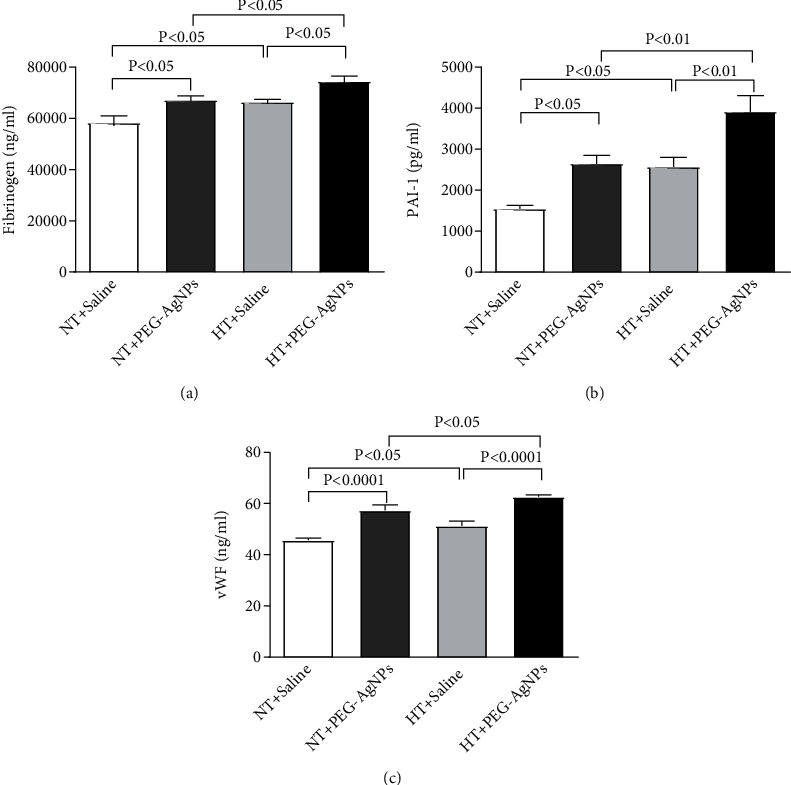
Fibrinogen (a), plasminogen activator inhibitor-1 (PAI-1 (b)), and von Willebrand factor (vWF (c)) concentrations in plasma, following intratracheal instillation of saline or polyethylene glycol silver nanoparticles (PEG-AgNPs) in normotensive (NT) or hypertensive (HT) mice. Data are the mean ± SEM (*n* = 6–8 in each group). Statistical analysis by one-way ANOVA followed by Holm-Sidak's multiple comparison test.

After exposure to PEG-AgNPs, the concentrations of fibrinogen were significantly increased in HT mice compared to either saline-exposed HT mice (*P* < 0.05) or PEG-AgNPs-exposed NT mice (*P* < 0.05) as represented in Figure 5(a).

The concentration of PAI-1 was significantly increased in PEG-AgNPs-exposed HT mice compared to either saline-exposed HT mice (*P* < 0.01) or PEG-AgNPs-exposed NT mice (*P* < 0.01) as represented in Figure 5(b).

A significant increase in the concentration of vWF was also seen in PEG-AgNPs-exposed HT mice compared to either saline-exposed HT mice (*P* < 0.0001) or PEG-AgNPs-exposed NT mice (*P* < 0.05) as represented in Figure 5(c).

In the Ag^+^ ions-exposed group, there was significant reduction in fibrinogen level in HT mice compared to either saline-exposed HT mice (*P* < 0.0001) and Ag^+^ ion-exposed NT mice (*P* < 0.0001). A significant reduction was also observed in concentration of PAI-1 in Ag^+^ ion-exposed HT mice compared to either saline-exposed HT mice (*P* < 0.05) and Ag^+^ ion-exposed NT mice (*P* < 0.01) (data shown in supplementary Figure S5).

### 3.6. Effect of PEG-AgNPs on SOD and NO

**Figure 6:**
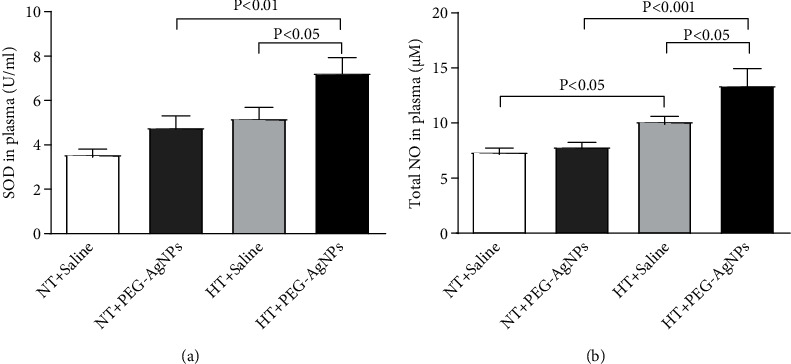
Superoxide dismutase (SOD (a)), total nitric oxide (NO (b)) levels in plasma, following intratracheal instillation of saline or polyethylene glycol silver nanoparticles (PEG-AgNPs) in normotensive (NT) or hypertensive (HT) mice. Data are the mean ± SEM (*n* = 6–8 in each group). Statistical analysis by one-way ANOVA followed by Holm-Sidak's multiple comparison test.

Figure 6 illustrates the effect of PEG-AgNPs on plasma levels of SOD and NO. The activity of SOD in plasma was significantly elevated in PEG-AgNPs-exposed HT mice compared to saline-exposed HT mice (*P* < 0.05) and PEG-AgNPs-exposed NT mice (*P* < 0.01) as shown in Figure 6(a).

Likewise, the level of NO was significantly increased in PEG-AgNPs-exposed HT mice compared to either saline-exposed HT mice (*P* < 0.05) or PEG-AgNPs-exposed NT mice (*P* < 0.001) as shown in Figure 6(b).

Ag^+^ ions caused significant elevation of SOD (*P* < 0.05) in HT mice compared to NT mice. A significant increase of NO was also observed in this group in Ag^+^ ions-exposed HT mice compared to either saline-exposed HT mice (*P* < 0.0001) or Ag^+^ ions-exposed NT mice (*P* < 0.0001) (data shown in supplementary Figure S6).

## 4. Discussion

Application of AgNPs has been increasing immensely over the last few years in various industries, given their strong antimicrobial properties [26, 34, 35]. Consequently, their potential exposure to environment and human health via various routes including respiratory, oral, and dermal also soared, warranting intensive studies in order to understand the safe application and effect of AgNPs on human health [36]. A considerable amount of studies has reported Ag accumulation and toxicity to local as well as distant organs following AgNPs exposure [37–39]. To this regard, several data revealed the toxic effects of different kinds of AgNPs (shaped, sized, coated, and functionalized) on the cardiovascular system including systemic inflammatory response, oxidative stress, DNA damage, apoptosis, and thrombosis [14, 15]. An aspect that has been overlooked is the nanotoxicity effects on susceptible populations as majority of the studies mainly focused on the risks to healthy adult population. Susceptible populations, due to alterations in physiological structure and functions, may suffer more damage and toxicity from the same exposure. A study by Holland et al. [16] revealed that pulmonary exposure to different size and coated AgNPs (size 20 nm and 110 nm, coating: polyvinylpyrrolidone and citrate) can induce exacerbation of cardiovascular injury such as expansion of cardiac/ischemic reperfusion injury. Recently, another investigation of cardiovascular responses to AgNPs (15 ± 4 nm) in spontaneously hypertensive rats suggested that hypertension intensified AgNPs cardiotoxicity [40].

Despite collection of studies reporting into how pulmonary exposure to AgNPs may impact cardiovascular toxicity, there are far fewer investigations on the impact of respiratory exposures on populations with preexisting cardiovascular conditions such as hypertension. In an effort to address these influences, we evaluated the effects of pulmonary-exposed AgNPs in healthy BALB/C mice and compared the same in a HT model. Our findings showed that PEG-AgNPs can induce acute dose-dependent cardiovascular effects including thrombosis, oxidative stress, and coagulation, and these effects were significantly aggravated in the animal model of hypertension. Due to potential of AgNPs to dissociate to Ag^+^ ions, we further studied the latter effects in NT and HT mice exposed to Ag^+^ ions.

In our present study, we used PEG-AgNPs and characterized them using TEM which revealed a homogenous particle size of 40 nm, correlating with the size of the manufacturer. Similar to our previous studies, intratracheal instillation was chosen to simulate pulmonary exposure which gave better control of the dose administered, given the fact that mice are obligate nose breathers and filter most inhaled particles. The dose selected was comparable to those in previous animal models of AgNPs exposure [41].

We have recently shown that single i.t. administration of AgNPs induced a significant dose-dependent shortening of the thrombotic occlusion time in pial arterioles and venules, 1 and 7 days after exposure, indicating that AgNPs possess prothrombotic effects [14]. Also, it is well known that thrombogenesis is the basic pathophysiological process underlying the major complications of hypertension [23, 42]. Interestingly, our current data reveals a marked shortening of thrombotic occlusion time in the HT mice compared to NT mice, confirming the prothrombotic effect of PEG-AgNPs. To gain further insights, into the mechanism underlying the latter effects of PEG-AgNPs, we investigated *in vitro* platelet aggregation in whole blood and the coagulation pathways. In agreement with our previous study [14], the addition of ADP into the whole blood of PEG-AgNPs-exposed NT mice caused significant platelet aggregation *in vitro*, and much stronger effect was observed in the whole blood of HT mice exposed to PEG-AgNPs. Furthermore, we demonstrated enhanced activation of PT and aPTT in HT mice compared to NT mice in response to AgNPs exposure. These effects reflect aggravated hypercoagulability caused by PEG-AgNPs in the HT model. We further studied the effects of PEG-AgNPs on haemostatic markers including fibrinogen, PAI-1, and vWF. Fibrinogen is an acute-phase protein that increases blood viscosity and promotes thrombus formation. Our data shows significant increase in fibrinogen levels in NT mice exposed to PEG-AgNPs, and the effect is aggravated in the HT mice. Likewise, an exacerbated effect was also observed with PAI-1, a potent endogenous inhibitor of fibrinolysis in HT mice comparted to NT mice receiving AgNPs. vWF is a biomarker for endothelial damage, and an increase in its level is associated with hypertension and cardiovascular disease [43]. Interestingly, our data reveals that this increase is further accelerated in PEG-AgNPs-exposed HT mice. Our results are in corroboration with previous *in vitro* and *in vivo* studies demonstrating AgNPs increase platelet aggregation, procoagulant activity, and consequently enhance thrombus formation [8, 14, 44, 45]. In line with our data, a recent study evaluating effect of titanium oxide nanoparticles exhibited significant deterioration of hemodynamic performance, demonstrated by an increase in the left ventricular end-diastolic pressure, decrement in the maximal rate of left ventricular pressure rise and decline, and marked prolongation of isovolumic contraction time in spontaneously hypertensive rats [46]. Further, our results corroborate findings that showed that exposure to particulate matter air pollution and nanoparticles is associated with changes in the global coagulation function suggesting a tendency toward hypercoagulability [21, 22, 47].

Similar to many nanoparticles, dissolution of PEG-AgNPs to Ag^+^ ions in vivo may also contribute to the cause of the observed effect discussed above. Hence, we repeated the identical tests on the Ag^+^ ion-exposed group. Interestingly, we found similar significant effects with regard to thrombotic occlusion time, PT, aPTT, and *in vitro* platelet aggregation. However, distinct results were obtained with the level of fibrinogen and PAI-1 and no effects were observed on the level of vWF. This dissimilar pattern of data observed corroborates with our previous study indicating Ag^+^ ions may induce different pathophysiological effect compared to the nanoparticle form [14, 48].

A central phenomenon associated with vascular structural and functional changes in hypertension, leading to cardiovascular disease, stroke, and renal failure, is oxidative stress, generating an imbalance between the levels of reactive oxygen species and antioxidants such as superoxide dismutase [20]. This condition also plays a key role in AgNPs-induced toxicity in living organisms [49–51]. In fact, the data in our present study shows an increase of superoxide dismutase in AgNPs-exposed NT mice compared to saline-exposed NT mice, and this effect was significantly aggravated in HT mice exposed to AgNPs or Ag^+^. Likewise, similar results were also obtained with the level of total NO. The increase of SOD and NO in our study indicates the development of counterbalance system that in turn prevents the potentially damaging activity of reactive oxygen species by antioxidant defence mechanisms [52, 53].

The limitations of the present work include the fact that we did not differentiate the observed effects in male and female mice separately. Given the fact that gender is an important biological variable in biomedical research and hormonal variations can potentially influence vascular function and circulating factors [54], additional studies are required to clarify this point. We evaluated the impact of pulmonary-exposed PEG-AgNPs on the model of hypertension. Nevertheless, it is equally important to understand the impact of AgNPs on other susceptible populations, for instance, diabetes, cancer, and pregnancy, before their wider use. Hence, additional research is warranted to assess AgNPs toxicity among other populations of increased susceptibility or disease model.

In conclusion, our present study provides a number of assertions regarding the correlation of the biological impact of AgNPs on models of hypertension and their possible mechanism. Our data reveals that pulmonary-exposed AgNPs can exacerbate procoagulatory and systemic oxidative stress in animals with preexisting hypertension. The findings of the present work have potential clinical significance. Actually, silver nanoparticles are widely used for therapeutic interventions and diagnosis in medical practice (e.g., drug carriers, nanoprobes, bioimaging, and labeling agents). In this regard, susceptible populations, due to alterations in physiological structure and functions, may suffer more damage and toxicity from the same exposure. Our data revealed that the impact of pulmonary exposure to silver nanoparticles is more severe in hypertensive animals compared to normotensive mice, indicating the importance of assessing the toxicity of nanoparticles not only in healthy people but also in populations with high risk factors or disease.

## Data Availability

The data that support the findings of this study are available from the corresponding author (Abderrahim Nemmar), upon reasonable request.

## Supplementary Materials

Supplementary 1 Figure S1: systolic blood pressure, measured prior to sacrifice and analysis. Data are the mean ± SEM (*n* = 8 in each group). Statistical analysis by unpaired Student's *t*-test.

Supplementary 2 Figure S2: thrombotic occlusion time in pial arterioles (A) or venules (B) following intratracheal instillation of saline or silver acetate (AgAc) in normotensive (NT) or hypertensive (HT) mice. Data are the mean ± SEM (*n* = 6–8 in each group). Statistical analysis by one-way ANOVA followed by Holm-Sidak's multiple comparison test.

Supplementary 3 Figure S3: prothrombin time (PT, A) and activated partial thromboplastin time (aPTT, B) measured following intratracheal instillation of saline or silver acetate (AgAc) in normotensive (NT) or hypertensive (HT) mice. Data are the mean ± SEM (*n* = 6–8 in each group). Statistical analysis by one-way ANOVA followed by Holm-Sidak's multiple comparison test.

Supplementary 4 Figure S4: in vitro platelet aggregation in whole blood collected from normotensive (NT) or hypertensive (HT) mice after intratracheal (i.t.) instillation of saline or silver acetate (AgAc). Blood samples obtained from the aforementioned groups were incubated at 37°C with ADP (0.1 *μ*M) for 3 min and stirred for another 3 min, and single platelets were then counted. The degree of platelet aggregation in HT or NT mice exposed to AgAc or saline was compared with each other and with that obtained in untreated (without ADP) whole blood obtained from control (unexposed) mice. Data are the mean ± SEM (*n* = 4). Statistical analysis by one-way ANOVA followed by Holm-Sidak's multiple comparison test.

Supplementary 5 Figure S5: fibrinogen (A), plasminogen activator inhibitor-1 (PAI-1, B), and von-Willebrand factor (vWF, C) concentrations in plasma, following intratracheal instillation of saline or silver acetate (AgAc) in normotensive (NT) or hypertensive (HT) mice. Data are the mean ± SEM (*n* = 6–8 in each group). Statistical analysis by one-way ANOVA followed by Holm-Sidak's multiple comparison test.

Supplementary 6 Figure S6: superoxide dismutase (SOD, A) and total nitric oxide (NO, B) levels in plasma, following intratracheal instillation of saline or silver acetate (AgAc) in normotensive (NT) or hypertensive (HT) mice. Data are the mean ± SEM (*n* = 6–8 in each group). Statistical analysis by one-way ANOVA followed by Holm-Sidak's multiple comparison test.
