# Supplementary figures and images for: Exacerbation of Thrombotic Responses to Silver Nanoparticles in Hypertensive Mouse Model
Source: Oxid Med Cell Longev. 2022 Jan 15;2022:2079630. doi: 10.1155/2022/2079630 (PMC8802099; doi:10.1155/2022/2079630)

Figure S1

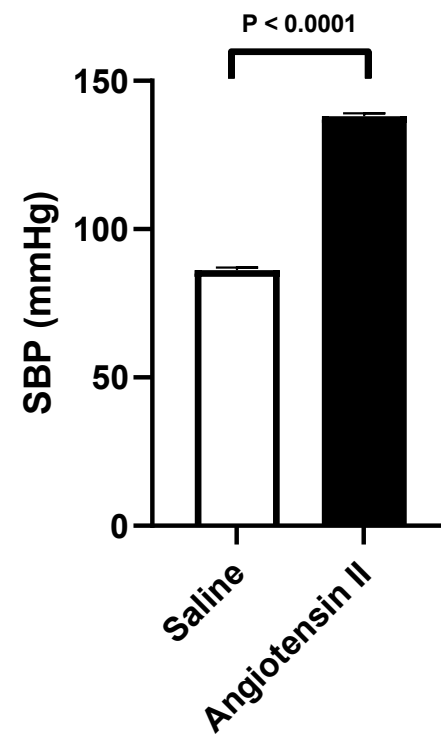

Supplement: Supplementary 1 — Figure S1: systolic blood pressure, measured prior to sacrifice and analysis. Data are the mean ± SEM (n = 8 in each group). Statistical analysis by unpaired Student's t-test. [file 2079630.f1.pdf]

Figure S2

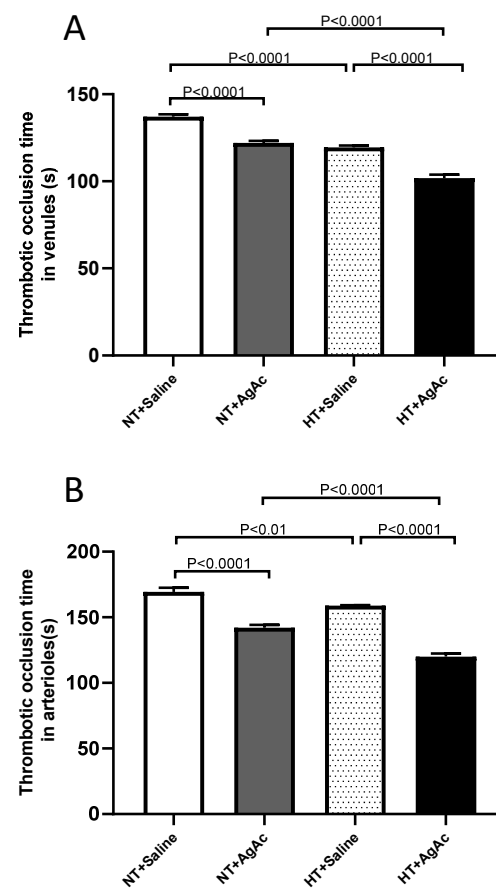

Supplement: Supplementary 2 — Figure S2: thrombotic occlusion time in pial arterioles (A) or venules (B) following intratracheal instillation of saline or silver acetate (AgAc) in normotensive (NT) or hypertensive (HT) mice. Data are the mean ± SEM (n = 6–8 in each group). Statistical analysis by one-way ANOVA followed by Holm-Sidak's multiple comparison test. [file 2079630.f2.pdf]

Figure S3

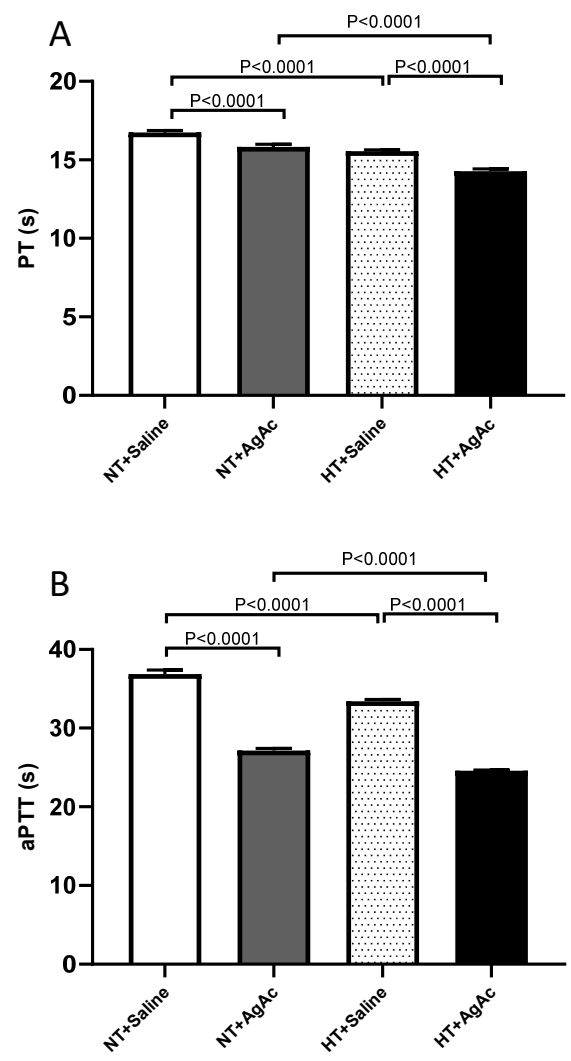

Supplement: Supplementary 3 — Figure S3: prothrombin time (PT, A) and activated partial thromboplastin time (aPTT, B) measured following intratracheal instillation of saline or silver acetate (AgAc) in normotensive (NT) or hypertensive (HT) mice. Data are the mean ± SEM (n = 6–8 in each group). Statistical analysis by one-way ANOVA followed by Holm-Sidak's multiple comparison test. [file 2079630.f3.pdf]

Figure S4

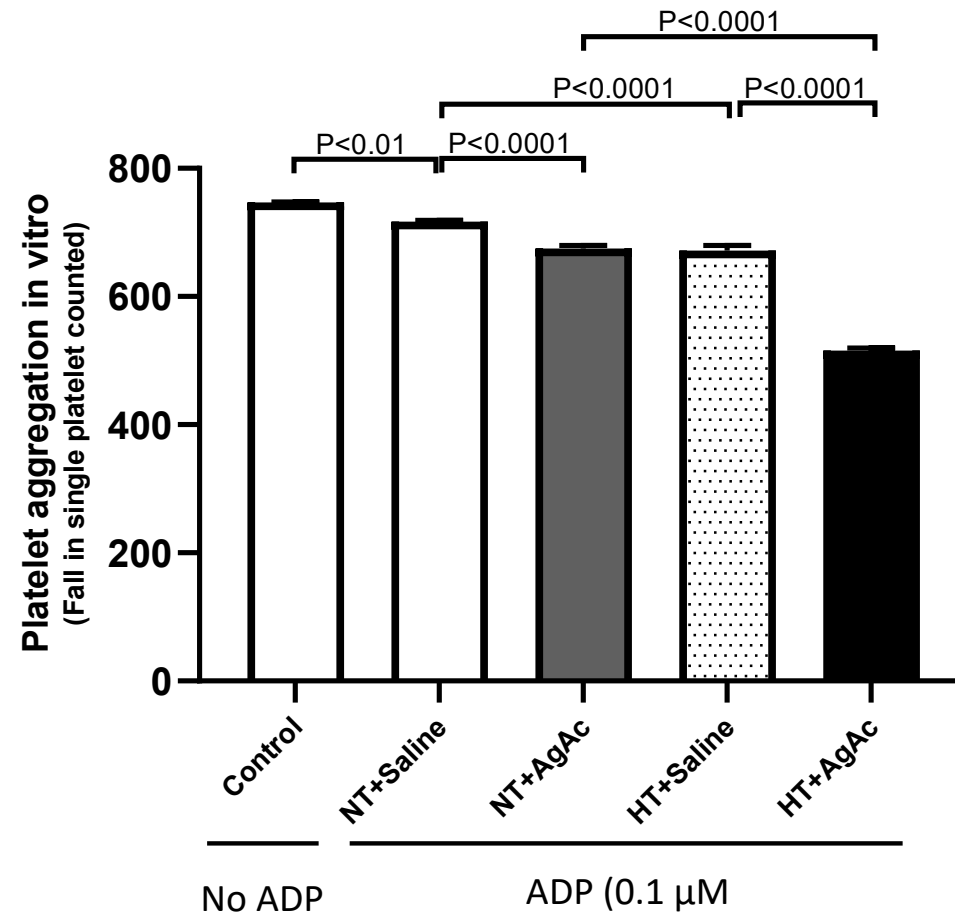

Supplement: Supplementary 4 — Figure S4: in vitro platelet aggregation in whole blood collected from normotensive (NT) or hypertensive (HT) mice after intratracheal (i.t.) instillation of saline or silver acetate (AgAc). Blood samples obtained from the aforementioned groups were incubated at 37°C with ADP (0.1 μM) for 3 min and stirred for another 3 min, and single platelets were then counted. The degree of platelet aggregation in HT or NT mice exposed to AgAc or saline was compared with each other and with that obtained in untreated (without ADP) whole blood obtained from control (unexposed) mice. Data are the mean ± SEM (n = 4). Statistical analysis by one-way ANOVA followed by Holm-Sidak's multiple comparison test. [file 2079630.f4.pdf]

Figure S5

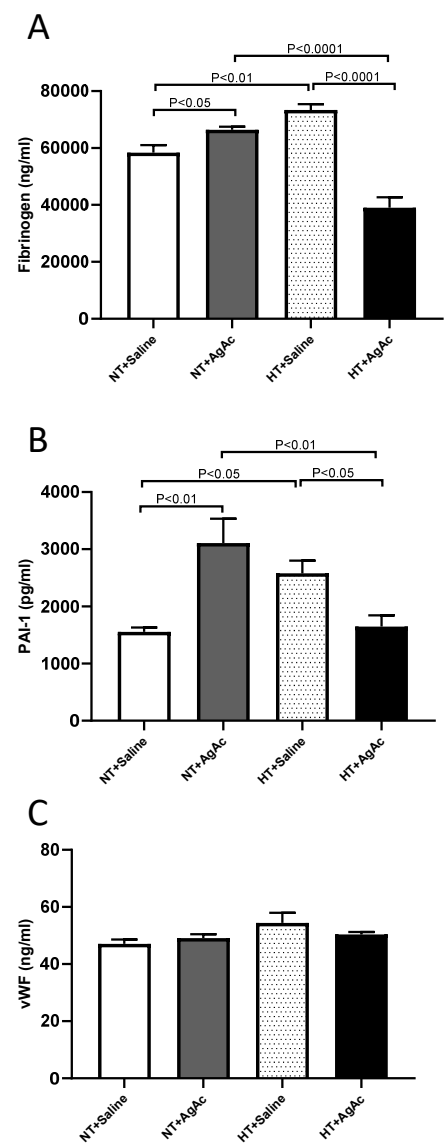

Supplement: Supplementary 5 — Figure S5: fibrinogen (A), plasminogen activator inhibitor-1 (PAI-1, B), and von-Willebrand factor (vWF, C) concentrations in plasma, following intratracheal instillation of saline or silver acetate (AgAc) in normotensive (NT) or hypertensive (HT) mice. Data are the mean ± SEM (n = 6–8 in each group). Statistical analysis by one-way ANOVA followed by Holm-Sidak's multiple comparison test. [file 2079630.f5.pdf]

Figure S6

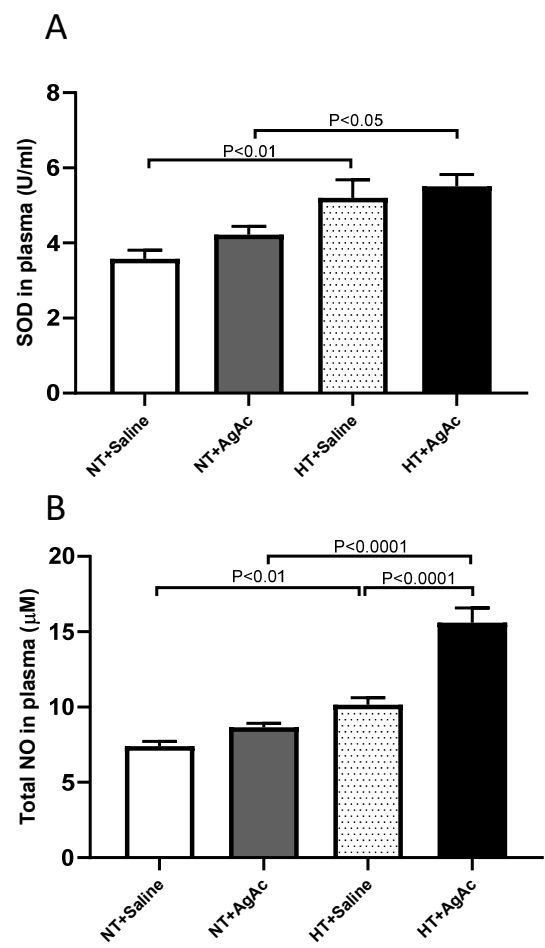

Supplement: Supplementary 6 — Figure S6: superoxide dismutase (SOD, A) and total nitric oxide (NO, B) levels in plasma, following intratracheal instillation of saline or silver acetate (AgAc) in normotensive (NT) or hypertensive (HT) mice. Data are the mean ± SEM (n = 6–8 in each group). Statistical analysis by one-way ANOVA followed by Holm-Sidak's multiple comparison test. [file 2079630.f6.pdf]
